# Effects of Oral Ingestion of L-Ornithine on Mental Stress and Fatigue Based on the Trier Social Stress Test in Healthy Humans: A Randomized, Double-Blind, Placebo-Controlled, Parallel-Group Trial

**DOI:** 10.3390/jcm13247583

**Published:** 2024-12-13

**Authors:** Kazuki Moriyasu, Atsushi Nakajima, Masahiko Morita

**Affiliations:** 1 Institute of Health Sciences, Kirin Holdings Company, Limited, Shonan Health Innovation Park 26-1-12-12 Muraoka-Higashi 2-chome, Fujisawa 251-8555, Japan; kazuki_moriyasu@kirin.co.jp; 2 Ueno-Asagao Clinic, 6F Kairaku Building, 2-7-5, Higashiueno, Taito-ku, Tokyo 110-0015, Japan; a.nakajima@ueno-asagao.clinic

**Keywords:** L-ornithine, mental stress, fatigue, Trier Social Stress Test, profile of the mood states

## Abstract

**Background:** With changes in the social environment typified by COVID-19, an increasing number of people are suffering from mental stress in interpersonal relationships and the resulting fatigue in recent years. L-ornithine oral ingestion reportedly suppresses the secretion of cortisol, a stress marker, through the hypothalamic–pituitary–adrenal (HPA) axis. However, there is insufficient research to determine whether L-ornithine exerts an ameliorative effect on social stress. Therefore, in this study, we investigated how L-ornithine affects mental stress and fatigue after social stress in healthy participants. **Methods:** We randomly assigned 65 participants into two groups, ingesting orally either 1600 mg of L-ornithine or a placebo for 7 days. On the day of the Trier Social Stress Test (TSST), participants took test products one hour before the testing. We evaluated the effects on saliva cortisol and mood states, including fatigue. **Results:** While L-ornithine did not affect saliva cortisol levels, it significantly improved the fatigue–inertia and anger–hostility scales of the Profile of Mood States on the morning after the TSST in the L-ornithine group compared to the placebo group. **Conclusions:** In conclusion, L-ornithine could potentially improve interpersonal social-stress-associated fatigue without involving the HPA axis. Trial registration: UMIN Clinical Trials Registry: UMIN000048949.

## 1. Introduction

Various recent changes have affected our social environment, such as the COVID-19 outbreak and the associated IT acceleration. In particular, during the COVID-19 epidemic, in-person interactions and conversations were significantly restricted, leading to anxiety or mental stress [1]. A survey involving 1000 Japanese employees in August 2021 during the COVID-19 epidemic revealed that short conversation times were strongly associated with poorer mental health compared to long conversation times [2]. Mental stress causes various disorders, such as fatigue or loss of vigor, and could finally lead to mental illness, such as depression [3]. Although the pandemic has recently come to an end and social activities have started to resume, we are facing new, emerging problems. A systematic review summarizing various post-pandemic retrospective studies described that widespread social isolation has a significant negative impact on post-pandemic mental health, especially among adolescents [4]. In fact, the mental health of college students reportedly declined during COVID-19 mitigation [5]. Various types of stressors could be distinguished, but in the given social background, the influence of psychosocial stressors (e.g., interpersonal relationships) will assumably increase in the future. Therefore, reducing the mental stress caused by psychosocial stressors could be clinically significant.

Mental stress reportedly causes various physiological responses, and one of the stress response pathways is the hypothalamic–pituitary–adrenal (HPA) axis [6]. Activation of the HPA axis by various stressors promotes the secretion of cortisol from the adrenal cortex, affecting the cardiovascular and metabolic system, immune functions, behavior, and reproduction [7]. Cortisol sensitively reflects the state of stress and has been used as a stress marker [8].

L-ornithine is a nonproteinogenic amino acid contained in various foods, although usually in very low amounts. Orally administered L-ornithine is reportedly uptaken into the portal vein from the intestines and delivered to various tissues [9]. The most famous clinical effect of L-ornithine is ammonia detoxification enhancement via urea cycle activation in the liver [10]. Orally administered L-ornithine reportedly suppressed corticosterone secretion during restraint stress in mice [11]. Moreover, several clinical studies have indicated that L-ornithine oral administration suppresses cortisol increase [12,13]. Concerning the underlying mechanisms, L-ornithine might exert an inhibitory effect on the HPA axis via the gamma-aminobutyric acid type A receptor (GABAA) [14]. Based on these findings, we hypothesized that L-ornithine oral administration could relieve stress and improve mental-stress-related fatigue and mood status.

To confirm how L-ornithine affects psychosocial stress, we performed a randomized, double-blind, placebo-controlled, parallel-group trial utilizing the Trier Social Stress Test (TSST). The TSST is a mental stress test to study human stress response [15]. This test has a feature wherein the subjects can be stressed face to face, and several studies have reported that it significantly increased the salivary cortisol levels [15,16]. It is thus suitable as a method to evaluate potential L-ornithine antistress effects.

## 2. Materials and Methods

### 2.1. Study Procedures

Our study protocol was approved by the Research Ethical Review Committee of Kirin Holdings Company, Limited (Tokyo, Japan), in accordance with the ethical standards established in the Helsinki Declaration and the ethical guidelines for epidemiological research of the Ministry of Education, Culture, Sports, Science, and Technology and the Ministry of Health, Labor, and Welfare in Japan. This study was registered with the UMIN Clinical Trials Registry as UMIN000048949, and it was conducted in compliance with the protocol. Written informed consent was obtained from all participants. This study was performed by a contract research organization, TES Holdings Co., Ltd. (Tokyo, Japan), from August 2022 to August 2023. Recruitment and intervention were conducted from September 2022 to December 2022 at Ueno-Asagao Clinic (Tokyo, Japan) and the TKP Ichigaya Conference Center (Tokyo, Japan).

### 2.2. Participants

In this study, we recruited healthy Japanese adults from the authorized volunteer bank for clinical trials. The entry criteria were as follows. (1) Men and women aged between 20 and 60 years at the time of obtaining consent to participate in the study. (2) Judged to be healthy by the investigator and with no chronic physical illness. (3) Prone to fatigue in daily life. (4) Feel stress, blush, heartbeat, etc. when speaking in front of people. (5) Able to be present in the office on the designated inspection date and undergo inspection. (6) Have received sufficient explanation about the purpose and contents of this study, voluntarily applied for participation after understanding the details of the ability to consent, and could consent to participate in this study in writing. (7) Deemed appropriate to participate in this study by the clinical investigator. The exclusion criteria were as follows. (1) Receiving drug treatment, or regularly using drugs for purposes other than treating a disease. (2) Suffering from a disease, undergoing treatment, or having a history of serious diseases, such as diabetes, kidney/liver disease, heart disease, thyroid disease, adrenal disease, or other metabolic diseases. (3) Undergoing treatment for psychological stress and using related or test product ingredient containing medicines, supplements, and health foods. (4) Regular antihistamine intake. (5) Being diagnosed with dry mouth or having subjective symptoms of it. (6) Having a score of at least 16 on the Quick Inventory of Depressive Symptomatology. (7) Being diagnosed with chronic fatigue syndrome or judged by the investigator or coordinator to have severe fatigue. (8) Having a history of drug dependence, alcohol dependence, or current medical history. (9) Potential allergy to the ingredients contained in the test food and potential serious allergy to other foods and pharmaceuticals. (10) Persons with severe anemia. (11) Persons with BMIs below 18.5 or beyond 25 kg/m^2^. (12) Expected or past life events with expected significant psychological impact during the study period. (13) Having a profession or experience in speaking in public. (14) Working for a company that develops, manufactures, and sells functional foods. (15) Smokers. (16) Troubles sleeping at night due to irregular work or other reasons. (17) Participation or foreseen participation in studies of ingesting foods, using drugs, and applying cosmetics and drugs during the study period. (18) Breastfeeding, being pregnant, potentially being pregnant, or planning pregnancy during the study period. (19) Judged by the clinical investigator to be unsuitable to participate.

### 2.3. Test Product

L-ornithine monohydrochloride was purchased from Kyowa Hakko Bio (Tokyo, Japan). We prepared two tablet types: one containing 1600 mg of L-ornithine per serving as an active tablet and the other containing microcrystalline cellulose per serving as a placebo. Table 1 summarizes the nutritional compositions of the test products. The study controller verified that there were no discernible differences in appearance, taste, and odor of the two test products prior to allocation.

### 2.4. Study Design

This study was a randomized, double-blind, placebo-controlled, parallel-group trial. The overall test schedule was approximately 8 weeks, consisting of Screening 1 (Visit 1), Screening 2 (Visit 2), ingestion, and TSST day (Visit 3). Screening 1 was conducted at the Ueno-Asagao Clinic. During Screening 1, we conducted measurements of anthropometric and circulatory parameters, hematology tests, blood biochemistry tests, urinalysis, and doctor interviews to confirm the health status. Additionally, we used the Japanese version of the Profile of Mood States second edition—Adult short (POMS2). POMS2 is an internationally well-established questionnaire for assessing mood states [17]. POMS-2 includes seven subscales (Anger–Hostility (AH), Confusion–Bewilderment (CB), Depression–Dejection (DD), Fatigue–Inertia (FI), Tension–Anxiety (TA), Vigor–Activity (VA), Friendliness (F)) and one summary scale (Total Mood Disturbance (TMD; sum of negative subscales minus vigor–activity)). The scores were converted to T scores with a mean equal to 50 and a standard deviation equal to 10. The FI T and VA T scores were ranked in order of high values and low values, respectively, and the top 106 rankings obtained by totaling the ranks of the two scores were assigned to Screening 2. We conducted Screening 2 and the TSST day at the TKP Ichigaya Conference Center. TSST was conducted in Screening 2 to select people with stress sensitivity. We collected a saliva sample at each time point and selected those with a salivary cortisol level increase of at least 2.5 nmol/L after 30 min of TSST compared to baseline.

The study controller performed a stratified randomization method using sex, age, baseline salivary cortisol concentration, and salivary cortisol Cmax value as stratification factors. The first allocation factor, sex, was divided into two strata for men and women, the second factor, age, was divided into two strata based on the median value, the third factor, baseline saliva cortisol concentration, was divided into two strata based on the median value, and the fourth factor, saliva cortisol Cmax, was divided into two strata based on the median value. The participants were randomly assigned using computer-generated random numbers. The assignment list was generated by the controller of the study. The two types of test products were assigned identification codes by another administrator, which were sealed by the administrator until the analysis data were fixed. Therefore, the study controller was unable to distinguish between the contents of the two test products. The assignment list was kept in a locked container, and all participants, investigators, and study personnel, except for the controller, were blinded to the assignment for the duration of the study. The dose of L-ornithine was 1600 mg per serving. Miyake et al. showed a significant decrease in the cortisol/DHEA-S ratio observed at L-ornithine doses of 400 mg/day at 4 weeks after the start of intake [13]. Conversely, this study had a shorter intake period of only 8 days. Because L-ornithine blood concentrations increase in a dose-dependent manner [18,19], a higher dose is likely necessary to achieve similar effects in a shorter period of time. Considering the time it took for the supplements to demonstrate efficacy in a previous study [13] and the administration period in this study, the administration dose was thus set at 1600 mg/day. Participants were instructed to consume the test products any time after breakfast during the 7-day intake period. On day 8 (Visit 3), the participants visited the TKP Ichigaya Conference Center and consumed the test products 1 h before the TSST. We collected saliva 45 and 2 min before the TSST and 1, 10, 20, 30, 60, and 120 min after the TSST. We conducted POMS2 45 min before the TSST, 30 and 120 min after the TSST, and the next morning (upon waking). A Visual Analog Scale (VAS) for fatigue assessment was conducted 45 and 2 min before the TSST and at 1, 10, 20, 30, 60, and 120 min after the TSST as well as the next morning. Participants marked their fatigue level on a 100 mm horizontal line (0 = not tired at all, 100 = extremely exhausted), with the distance from the left end of the line measured as the VAS score.

**Figure 1:**
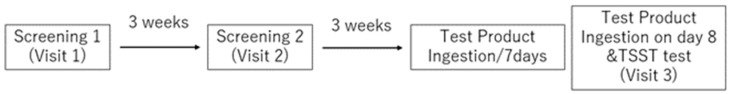
Overall test schedule. TSST, Trier Social Stress Test.

Participants were instructed to continue their usual eating, exercise, sleeping, and drinking habits throughout the study. The consumption of medicines, supplements, and health foods that contain ornithine or are related to mental stress was prohibited. Activities to relieve stress and recover from fatigue were prohibited (e.g., travel, hot spring, entertainment appreciation, karaoke, etc.). Job interviews and public speaking were prohibited during the test period. Participants were prohibited from drinking alcohol on the day before the test and were asked to fast for 12 h before the visit. After the visit, eating and drinking (except for the prepared standardized meal and water) were prohibited until the completion of testing at Visit 3. Smoking and drinking alcohol were also prohibited on the test day until after the POMS survey was completed the next morning. The overall study schedule is shown in Figure 1.

### 2.5. Sample Size Culculation

We used G*Power 3.1.9.2 to calculate the sample size. Using the saliva cortisol data from our previous similar study [20], the effect size was 0.833. Assuming the same effect size, 5% significance level, and 80% power, the required sample size was 19 per group. Considering that the protocol, especially the intake dose of the test product, was different from the previous study, and several clinical trials using the TSST to evaluate the effectiveness of foods had approximately 25–30 participants per group [16,21,22], the required number of participants was set at 30 per group.

### 2.6. TSST

We conducted the TSST according to previous reports [15]. To minimize the stress load variation among the participants, actors with technical training were employed to play the role of the TSST interviewer. The combination of participants and interviewers in Visit 2 and Visit 3 was changed to avoid the effect of habituation. Cortisol exhibits a characteristic diurnal fluctuation in that it rises rapidly when waking up, then declines, and remains relatively stable at a low level from the afternoon onward [23]. Therefore, we completed all saliva collections between 12:00 and 18:00 to minimize the circadian rhythm effect of cortisol.

### 2.7. Anthropometric and Circulatory Parameter Measurements

We measured the body weight, body fat ratio, systolic blood pressure, diastolic blood pressure, and heart rate at all Visits and height at Screening 1, and we calculated the BMI from the height and body weight.

### 2.8. Hematology Tests, Blood Biochemistry Tests, and Urinalysis

Blood and urine samples were collected at Screening 1. Hematology tests included white blood cell count, red blood cell count, hemoglobin, hematocrit, mean corpuscular volume, mean corpuscular hemoglobin concentration, and platelet count. Blood biochemistry tests included total protein, albumin, blood urea nitrogen, creatinine, uric acid, aspartate aminotransferase, alanine aminotransferase, gamma-glutamyl transferase, alkaline phosphatase, lactate dehydrogenase, creatine kinase, C-reactive protein, total cholesterol, triglycerides, low-density lipoprotein cholesterol, high-density lipoprotein (HDL) cholesterol, non-HDL cholesterol, total bilirubin, sodium, potassium, chloride, calcium, magnesium, iron, glucose, and hemoglobin A1c. All samples were analyzed using standard methods at Hoken Kagaku Inc. (Kanagawa, Japan).

### 2.9. Endpoints

The primary endpoint was the salivary cortisol level after TSST. The secondary endpoints included fatigue and mood status, assessed using POMS2 after TSST. VAS was also used to evaluate fatigue as a secondary endpoint. Safety endpoints were any occurrence of adverse events. For saliva cortisol levels, in addition to the changes over time, the area under the curve (AUC) was also calculated using the trapezoidal method. Adverse events were determined by the clinical investigator based on inquiries and participant diaries. When an adverse event occurred, we conducted a follow-up survey until the event disappeared or recovered again.

### 2.10. Measurements of Saliva Cortisol

We collected the saliva samples using a Salimetrics Oral Swab (SOS) (SALIMETRICS, Carlsbad, CA, USA) and then divided the samples and kept them frozen at −80 °C until measurement at LSI Medience Corporation (Tokyo, Japan). We measured the saliva cortisol concentration with commercially available kits according to the manufacturer’s instructions (Salivary Cortisol Enzyme Immunoassay Kit, Salimetrics).

### 2.11. Statistical Analysis

The data were expressed as the mean ± SEM. We expressed only baseline characteristic data as the mean ± SD. All analyses were conducted in the per-protocol-set group. However, only the safety endpoint evaluation was conducted in the intention-to-treat group. We used Dunnett’s test to evaluate the change from the data at −45 min for salivary cortisol data and used unpaired *t*-tests to compare the difference between the two groups at each time point for salivary cortisol data. Additionally, an unpaired *t*-test was used for two-group comparisons of saliva cortisol AUC. We used the Mann–Whitney U test to compare the differences between the two groups at each time point for POMS2 and VAS data. We conducted the statistical analyses using SAS (SAS 9.4) and SPSS (Statistics26). *p* values less than 0.05 were considered to be statistically significant.

## 3. Results

### 3.1. Flow Diagram of Participant Progress and Background

**Figure 2:**
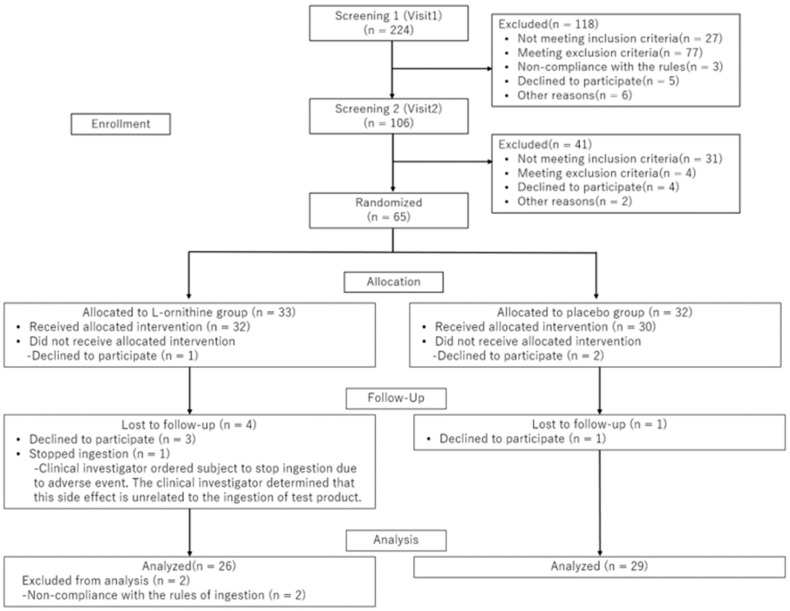
Flow diagram of the progression through the phases of the parallel randomized trial of two groups.

A flow diagram represents the progress of this study (Figure 2). Throughout the two-stage screening, 159 of 224 participants who consented to take part in the study were excluded, and 65 were randomly assigned to either L-ornithine or a placebo group. Another 3 participants declined to participate after allocation, and 62 participants started to take the test products. Four participants declined to participate during the ingestion period, and one participant was ordered to stop ingestion by the clinical investigator due to adverse events. However, the clinical investigator determined that this adverse event was unrelated to the ingestion of the test product. Fifty-seven participants completed the intervention, but two were excluded from the analysis because they violated the rules of supplementation protocol. The baseline characteristics of the 65 participants are shown in Table 2. Body weight, body fat ratio, systolic blood pressure, diastolic blood pressure, heart rate, and saliva cortisol data shown in Table 2 were collected in Screening 2. The other data shown in Table 2 were collected in Screening 1.

### 3.2. Primary Endpoint

**Figure 3:**
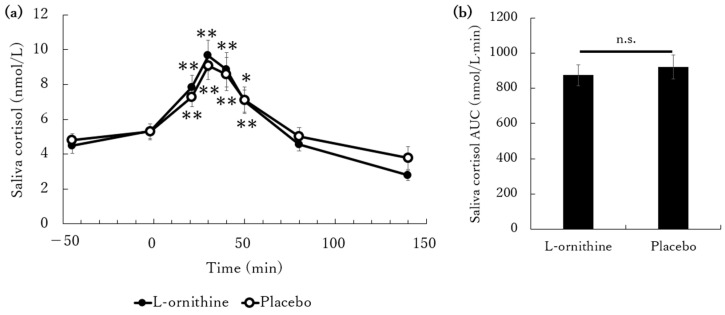
Time course of changes in saliva cortisol following the TSST. The subjects received either 1600 mg of L-ornithine or a placebo for 7 days. On the 8th day, the subjects took test products one hour before testing, and saliva cortisol was evaluated before and after the TSST. The TSST itself took 20 min. (**a**) Effects of L-ornithine or placebo on saliva cortisol. Saliva cortisol concentrations were measured at −45, −2, 21, 30, 40, 50, 80, and 140 min. (**b**) Area under the curve (AUC) of saliva cortisol. The data are expressed as the means ± SEM. *: *p* < 0.05, **: *p* < 0.01, vs. −45 min, n.s.: not significant.

Changes in saliva cortisol concentration are shown in Figure 3a. The basal saliva cortisol concentrations are the same between the two groups and significantly increased in both groups after conducting the TSST when compared with the baseline (−45 min). The cortisol level peaked at 30 min and then declined gradually. This characteristic transition was also observed in Screening 2. However, no significant differences were shown between the groups at any time point. Figure 3b shows the AUC for saliva cortisol. No significant differences were observed between the groups.

### 3.3. Secondary Endpoint

**Figure 4:**
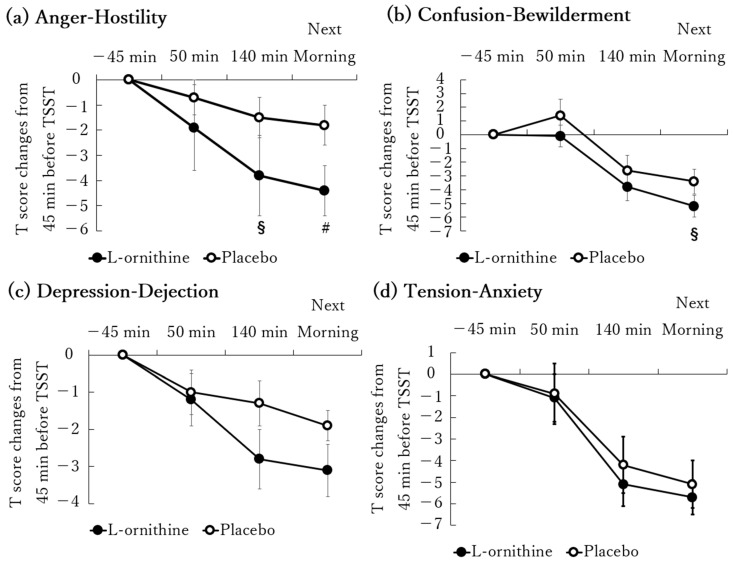

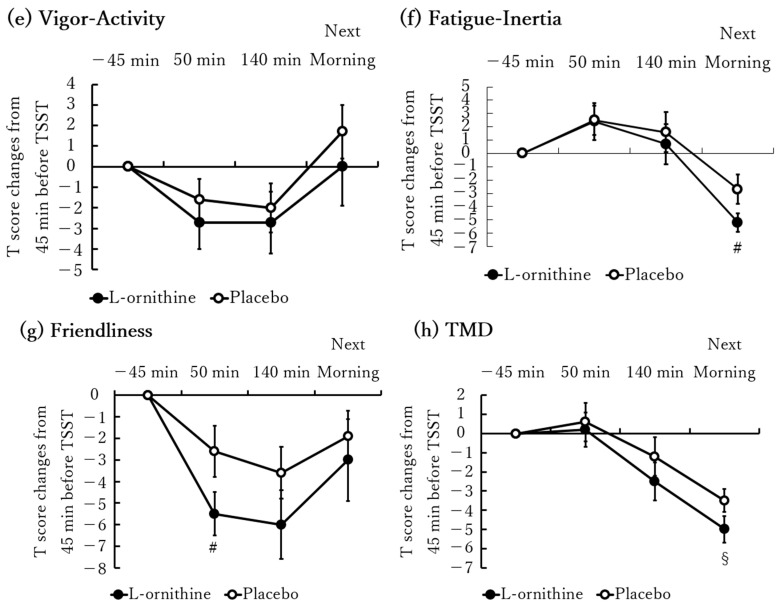
Time course of changes in POMS2 following the TSST. The subjects received either 1600 mg of L-ornithine or a placebo for 7 days. On the 8th day, the subjects took test products one hour before testing, and POMS2 was evaluated before and after the TSST and the next morning. The TSST itself took 20 min. Changes in T score of POMS2 from 45 min before TSST were shown. (**a**) AH, (**b**) CB, (**c**) DD, (**d**) TA, (**e**) VA, (**f**) FI, (**g**) F, (**h**) TMD. Data are expressed as means ± SEM. §: *p* < 0.10, #: *p* < 0.05, vs. placebo.

Changes in POMS2 T scores are shown in Figure 4. The T score of AH tended to improve in the L-ornithine group compared to the placebo group at 140 min and was significantly improved the next morning (Figure 4a). The T score of CB tended to improve in the L-ornithine group compared to the placebo group the next morning (Figure 4b). No significant differences were observed in the T scores for DD, TA, and VA between groups at any time point (Figure 4c–e). The T score of FI was significantly improved in the L-ornithine group compared to the placebo group the next morning (Figure 4f). The T score of F significantly decreased in the L-ornithine group compared to the placebo group at 50 min (Figure 4g). The T score of TMD, which comprehensively represents a negative mood state, tended to improve in the L-ornithine group compared to the placebo group the next morning (Figure 4h).

No significant differences in VAS score changes were observed between the L-ornithine and placebo groups at any time point (Figure S1).

### 3.4. Safety Endpoints

Safety endpoint evaluation was conducted on all participants who ingested test products at least once. Four adverse events, including skin disorders, gastrointestinal disorders (two cases), and infectious symptoms, were reported during the study. One out of the four participants (skin disorder) was ordered to stop ingestion by the clinical investigator, and the others continued to participate because of a slight level of transient symptoms. All adverse events were judged to have no relation to the test products by the clinical investigator.

## 4. Discussion

In this study, we evaluated how oral L-ornithine could affect mental stress and fatigue based on stress load. We adopted the TSST as the stress loading method developed by Kirschbaum et al. for evaluating the cortisol response to mental stimulation in healthy participants [15]. The TSST is frequently used to evaluate how food affects stress [21,22]. Therefore, it is considered ideal for evaluating the effects of oral administration of L-ornithine oral. In this study, saliva cortisol significantly increased based on the TSST and then decreased to a baseline level. This trend of cortisol values was in good agreement with those of previous studies [15,16], indicating that we conducted our study appropriately for mental stress impact evaluation.

We observed no significant differences in saliva cortisol levels between the groups at any time point, similarly to the results obtained for AUC. Previous studies revealed that orally administered L-ornithine is transported into the brain [11], and it suppresses corticosterone secretion under stressful conditions via GABAA receptors [12]. Moreover, several studies demonstrated that orally administered L-ornithine suppresses cortisol increase [12,13]. Therefore, the antistress effect of L-ornithine could be conditionally replicated. A potential reason for the lack of the expected results in this study could be the small amount of doses. In a similar previous study that we conducted, we evaluated the stress state during the TSST by ingesting 2400 mg of L-ornithine and described that L-ornithine had a suppressive effect on cortisol increase [20]. Because the dose administered in this study was 1600 mg, it could be potentially insufficient for the strong TSST stress stimulus. In fact, because the blood concentration of L-ornithine is dose-dependent [18,19], a smaller dose would yield a smaller increase in L-ornithine levels. In the same way, GABA, which is thought to exert an antistress effect via the HPA axis (similar to L-ornithine) [24], has dose-dependent antistress and antifatigue effects during mental stress [25], which may suggest the presence of a dose response for L-ornithine. Furthermore, unlike the previous study [20], the participants who were feeling fatigue were recruited in this study, which may have affected the result. Another possible reason might be the influence of the participant stress sensitivity. No medical standard value exists for saliva cortisol levels. Therefore, in this study, based on Kirshbaum et al.’s study [15], we set the screening conditions at an increase of at least 2.5 nmol/L at 30 min after the TSST, with the baseline being 45 min before the TSST. Kirschbaum et al. reported that more than 70% of their participants subjected to mental stress by the TSST exhibited increased saliva cortisol levels from the baseline of at least 2.5 nmol/L [15]. In this study, 56% of those who underwent mental stress through the TSST met the same criterion, which means that our subjects were not overly restricted in the general population. Saliva cortisol is a highly reliable and well-established index that reflects stress. However, for some reason, certain people display no cortisol-level-related changes in response to stress. Because verifying test product efficacy is difficult when targeting people who do not respond to stress, we set a reference value for participant enrollment with a response to stress in this study. Nevertheless, in this study, the participant number exceeding the specified value (increase of at least 2.5 nmol/L at 30 min following the TSST) was below the planned number, and the participants were thus enrolled in descending order of cortisol increase. As a result, we decided to include participants who did not reach the reference value with a low cortisol level increase after the TSST, which might have made the detection of the differences between the groups difficult.

However, we obtained interesting POMS2-related results. Changes in T scores in the AH and FI scales the next morning were significantly lower in the L-ornithine group than in the placebo group. At the same time point, the TMD T scores also tended to improve in the L-ornithine group compared to the placebo group. On the other hand, no significant differences between the L-ornithine and placebo groups were observed in VAS scores at the time points where significant differences in POMS2 FI scores were observed between groups. Although both VAS and POMS FI scales were used to assess the same concept of “fatigue”, the results from these scales do not always align. Brunier et al., for instance, reported that although a significant correlation was found between VAS and POMS scores when fatigue was evaluated by the same patients at the same time, the amount of shared variance was limited due to differences in the descriptive phrases used for each scale, showing the medians and distributions were different among the two fatigue variables [26]. There is ongoing debate among researchers regarding which scale is more appropriate for evaluating fatigue, with Brunier et al. suggesting that the multi-item POMS subscale may be more multidimensional and, therefore, a more comprehensive measure of fatigue than the unidimensional VAS [26]. Additionally, it has been suggested that using VAS alone to evaluate multidimensional indicators, such as pain, may not be recommended because of its unidimensional format [27]. Also, POMS2 is an internationally well-established questionnaire, especially for assessing mood states [17]. Based on these findings, it was thought that POMS2 FI may better reflect the subjects’ fatigue with higher reliability than VAS. We therefore considered the mechanism by which L-ornithine may relieve fatigue. Initially, we assumed that L-ornithine oral administration would improve fatigue and mood state by alleviating mental stress, but because the stress markers were unaffected in our study, we believe that the effect was exerted through a different mechanism. Aside from its stress-suppressing effect via the HPA axis, L-ornithine is known to have a wide range of functions, including ammonia detoxification by activating the urea cycle in the liver [10] and promoting growth hormone secretion [28] and wound healing by promoting collagen synthesis [29]. Among the various functions of L-ornithine, its ammonia detoxification effect is likely the most closely related to fatigue, although further studies are needed to confirm this. Ammonia is highly toxic to the human body, causing neurotoxicity, and it reportedly reduces tricarboxylic Acid cycle function due to α-ketoglutarate reduction, inhibited energy production, and induced fatigue [30,31,32]. Therefore, L-ornithine might improve fatigue by suppressing the increase in ammonia. The ammonia-fatigue theory has been proposed for a long time. Substantial indirect evidence has suggested their close relationship, such as frequent reports of fatigue in patients with hepatic encephalopathy primarily caused by hyperammonemia. Fatigue during exercise is also accompanied by an increase in blood ammonia [31,33,34]. In addition, Wilkinson et al. confirmed that intravenous administration of ammonium chloride in humans induced fatigue, further supporting the strong correlation between ammonia and fatigue [35]. Another report found that mental stress increases the rate of dissipation of ammonia from the skin in humans [36], which is believed to be derived from blood ammonia [36], suggesting that mental stress may increase the concentration of ammonia in the blood. Moreover, another study revealed that exposure to strong mental and physical stress causes a marked increase in blood ammonia in humans [37]. Although mental and physical stresses were combined in the previous study [37], there is a possibility that psychological stress loading may contribute to elevation of ammonia in blood. Observational studies of prisoners also presented that prisoners displayed higher depression and anxiety scores coupled with poorer mental health compared to the controls. Furthermore, blood ammonia levels were higher in the prisoner than in the control group, suggesting a relationship between mental health and blood ammonia levels, although the causal relationship remains unclear [38]. Further research would be required to unravel the mechanism underlying mental-stress-related blood ammonia increase, although a potential explanation is that stressors could potentially reduce liver function. Corticotropin-releasing factor (CRF) was actively secreted in the brains of rodents due to restraint stress or mental stress loading in previous in vivo studies [39,40]. Stressor-related CRF secretion in the brain leads to reduced liver blood flow through the sympathetic nerves, thereby lowering liver function [41,42]. Because ammonia is mainly detoxified by the ornithine cycle in the liver, the ability to metabolize ammonia likely decreases as the metabolic function of the liver is impaired. However, ornithine reportedly reduces blood ammonia concentration and alleviates fatigue by activating the ornithine cycle [43]. These reports suggest that L-ornithine probably exerts its antifatigue and mood-improving effects by suppressing the mental-stress-induced increased blood ammonia levels. Although the present study protocol is optimized to evaluate the antistress effects of L-ornithine, considering that mental stress load could increase ammonia levels [36] and that ammonia causes fatigue, we believe that our current protocol was also appropriate for evaluating fatigue. Furthermore, anger suggestibly appears as a characteristic emotional burden under overwork conditions, such as long-time intensive work, and a strong relationship is inferred between fatigue and anger [44]. The improvement in fatigue could have resulted in improvement of the AH-scale-related T score. The AH scale measures feelings of distress and hostility, expressed in terms of “Annoyed” or “resentful” [45]; therefore, lower AH scores may reflect a calm feeling.

The limitations of this study must be raised as well. First, no significant difference was observed between the groups in salivary cortisol levels, the primary endpoint, and no objective evidence was presented to support the fatigue-reducing effect of L-ornithine. We proposed the ammonia hypothesis as an alternative mechanism, but we did not analyze the blood ammonia profiles after strong psychosocial stress. This remains a hypothesis. Although some studies have suggested that mental stress could increase blood ammonia [37,38], the psychiatric sensitivities generally vary, and the kind of psychological stress that may be linked to notable changes in body ammonia level remains only partially verified. In particular, there are no reports about increased blood ammonia after the TSST. Therefore, further studies would be required to assess the mechanism by which elevated blood ammonia could be linked to mental stress in this TSST condition. Additionally, given the differences between VAS and POMS2 FI scores, further investigation would be warranted to determine whether L-ornithine has an antifatigue effect under conditions of severe mental stress.

All adverse events reported during the study period were judged to be mild and not related to the test products by the clinical investigator. Therefore, we considered 1600 mg L-ornithine intake safe for 8 days.

## 5. Conclusions

Taken together, our study revealed that 1600 mg of L-ornithine intake did not suppress saliva cortisol elevation against acute mental stress, although it could improve fatigue and anger after mental stress load in healthy humans. Further research would be required to determine the sufficient L-ornithine dosage to exert antistress effects and to demonstrate the underlying mechanism involved in mental stress and ammonia’s influence.

## Figures and Tables

**Table 1:** Nutritional composition of the test products (per serving).

	L-Ornithine Product	Placebo Product
Energy (kcal)	7.3	0.56
Protein (g)	1.685	0
Lipid (g)	0.06	0.06
Carbohydrate (g)	0.17	2.263
Sodium (g)	0	0
L-ornithine (mg)	1600	0

**Table 2:** Allocated participant baseline characteristics.

	L-Ornithine	Placebo
n	33	32
Age (years)	37.7 (10.5)	37.2 (12.0)
Sex (male)	16	15
Height (cm)	164.3 (8.7)	165.2 (9.0)
Weight (kg)	57.4 (6.9)	60.0 (9.0)
BMI (kg/m^2^)	21.2 (1.5)	21.9 (1.8)
Body fat ratio (%)	24.0 (6.4)	25.2 (6.0)
Systolic blood pressure (mmHg)	116.7 (11.7)	114.7 (11.4)
Diastolic blood pressure (mmHg)	73.0 (8.5)	71.1 (8.1)
Heart rate (bpm)	69.7 (6.1)	71.8 (8.6)
Saliva cortisol (nmol/L)	3.8 (1.6)	3.6 (1.3)
Saliva cortisol (nmol/L) (Cmax)	12.2 (7.1)	11.7 (7.4)
WBC (per μL)	4987.9 (1222.7)	5218.8 (1338.7)
RBC (per μL)	472.6 (42.4)	479.2 (48.1)
HGB (g/dL)	14.2 (1.3)	14.2 (1.7)
HCT (%)	44.1 (3.7)	44.6 (4.7)
MCV (fl)	93.5 (3.9)	93.4 (5.4)
MCH (pg)	30.1 (1.3)	29.6 (2.3)
MCHC (%)	32.2 (0.9)	31.7 (1.0)
PLT (X104/μL)	26.3 (4.9)	27.8 (6.2)
TP (g/dL)	7.3 (0.4)	7.2 (0.3)
ALB (g/dL)	4.7 (0.3)	4.7 (0.3)
BUN (mg/dL)	12.3 (3.4)	11.5 (3.1)
CRE (mg/dL)	0.8 (0.2)	0.8 (0.2)
UA (mg/dL)	5.1 (1.4)	5.0 (1.4)
AST (U/L)	19.4 (4.7)	19.7 (4.3)
ALT (U/L)	16.6 (7.0)	17.9 (8.4)
γ-GT (U/L)	22.7 (14.9)	21.2 (12.1)
ALP (U/L)	62.5 (18.3)	58.5 (11.8)
LD (U/L)	167.7 (27.7)	162.6 (23.5)
CK (U/L)	101.5 (35.7)	112.5 (45.0)
CRP (mg/dL)	<0.1	<0.1
T-CHO (mg/dL)	205.5 (33.8)	205.0 (32.7)
TG (mg/dL)	84.1 (73.0)	79.5 (50.9)
LDL cholesterol (mg/dL)	117.1 (25.2)	114.6 (26.8)
HDL cholesterol (mg/dL)	68.4 (17.9)	71.0 (17.1)
Non-HDL cholesterol (mg/dL)	137.0 (28.2)	134.0 (33.4)
T-BIL (mg/dL)	0.8 (0.3)	0.7 (0.2)
Na (mmol/L)	139.9 (2.2)	139.8 (1.7)
K (mmol/L)	4.3 (0.3)	4.3 (0.2)
Cl (mmol/L)	104.3 (1.9)	104.3 (2.3)
Ca (mg/dL)	9.4 (0.4)	9.3 (0.3)
Mg (mg/dL)	2.3 (0.2)	2.2 (0.2)
Fe (μg/dL)	107.7 (33.7)	100.3 (46.8)
GLU (mg/dL)	84.5 (5.2)	84.6 (6.6)
HbA1c (NGSP) (%)	5.1 (0.2)	5.2 (0.2)

Values are mean ± SD. BMI, body mass index; WBC, white blood cell count; RBC, red blood cell count; HGB, hemoglobin; HCT, hematocrit; MCV, mean corpuscular volume; MCH, mean corpuscular hemoglobin; MCHC, mean corpuscular hemoglobin concentration; PLT, platelet count; TP, total protein; ALB, albumin; BUN, blood urea nitrogen; CRE, creatinine; UA, uric acid; AST, aspartate aminotransferase; ALT, alanine aminotransferase; γ-GT, gamma-glutamyl transferase; ALP, alkaline phosphatase; LD, lactate dehydrogenase; CK, creatine kinase; CRP, C-reactive protein; T-CHO, total cholesterol; TG, triglycerides; LDL, low-density lipoprotein; HDL, high-density lipoprotein; T-BIL, total bilirubin; Na, sodium; K, potassium; Cl, chloride; Ca, calcium; Mg, magnesium; Fe, iron; GLU, glucose; HbA1c (NGSP), hemoglobin A1c (National Glycohemoglobin Standardization Program).

## Data Availability

The datasets generated and/or analyzed during the current study are not publicly available but are available from the corresponding author upon reasonable request wherever legally and ethically possible.

## Supplementary Materials

The following supporting information can be downloaded at: https://www.mdpi.com/article/10.3390/jcm13247583/s1, Figure S1: Time course of changes in VAS score following the TSST. The subjects received either 1600 mg of L-ornithine or a placebo for 7 days. On the 8th day, the subjects took test products one hour before testing, and VAS was evaluated before and after the TSST and the next morning. The TSST itself took 20 min. Changes in VAS score from 45 min before TSST were shown. No significant differences were observed between L-ornithine and placebo groups at any time point. Data are expressed as means ± SEM.
