# Supplementary material for: Effects of Oral Ingestion of L-Ornithine on Mental Stress and Fatigue Based on the Trier Social Stress Test in Healthy Humans: A Randomized, Double-Blind, Placebo-Controlled, Parallel-Group Trial
Source: J Clin Med. 2024 Dec 13;13(24):7583. doi: 10.3390/jcm13247583 (PMC11676746; doi:10.3390/jcm13247583)
Supplement: Supplementary file 1 [file jcm-13-07583-s001.zip › jcm-3326170-supplementary.pdf]

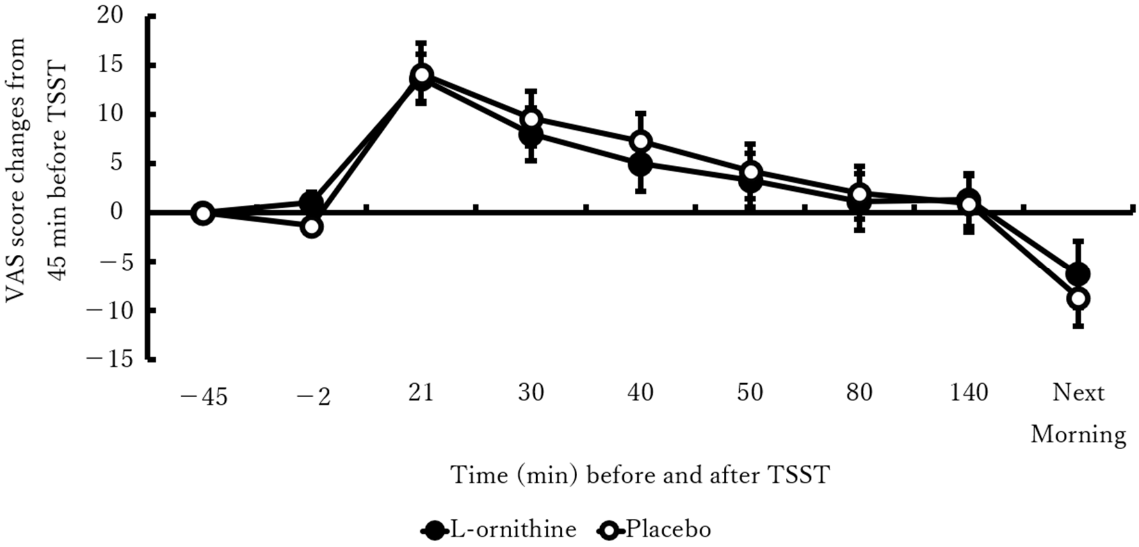

Figure S1. Time course of changes in VAS score following the TSST. The subjects received either 1600 mg of L-ornithine or a placebo for 7 days. On the 8th day, the subjects took test products one hour before testing, and VAS was evaluated before and after the TSST and the next morning. The TSST itself took 20 min. Changes in VAS score from 45 min before TSST were shown. No significant differences were observed between L-ornithine and placebo groups at any time point. Data are expressed as means ± SEM.
